# Addendum: The efficacy and safety of Favipiravir in treatment of COVID-19: a systematic review and meta-analysis of clinical trials

**DOI:** 10.1038/s41598-022-05835-2

**Published:** 2022-02-01

**Authors:** Soheil Hassanipour, Morteza Arab-Zozani, Bahman Amani, Forough Heidarzad, Mohammad Fathalipour, Rudolph Martinez-de-Hoyo

**Affiliations:** 1 Gastrointestinal and Liver Diseases Research Center, Guilan University of Medical Sciences, Rasht, Iran; 2 Social Determinants of Health Research Center, Birjand University of Medical Sciences, Birjand, Iran; 3 Department of Health Management and Economics, School of Public Health, Tehran University of Medical Sciences, Tehran, Iran; 4 Department of Pharmacology and Toxicology, Faculty of Pharmacy, Hormozgan University of Medical Sciences, Bandar Abbas, Iran; 5 MSN Labs Americas, Bogotá, Colombia

Addendum to: *Scientific Reports* https://doi.org/10.1038/s41598-021-90551-6, published online 26 May 2021

In the original version of this Article we included the data reported in a preprint by Dabbous et al. entitled "Safety and efficacy of favipiravir versus hydroxychloroquine in management of COVID-19: A randomised controlled trial", which was later published^1^. After publication of our Article it was brought to our attention that Dabbous et al.’ paper had subsequently been retracted^2^. We have therefore repeated sub-analyses in which this study was originally included to establish the effect of the removal of this data from the review.

The results of the analyses reported in the original Figures 3 and 7, updated for removal of Dabbous et al. data, are shown below (Figure 1 and 2, respectively).

**Figure 1:**
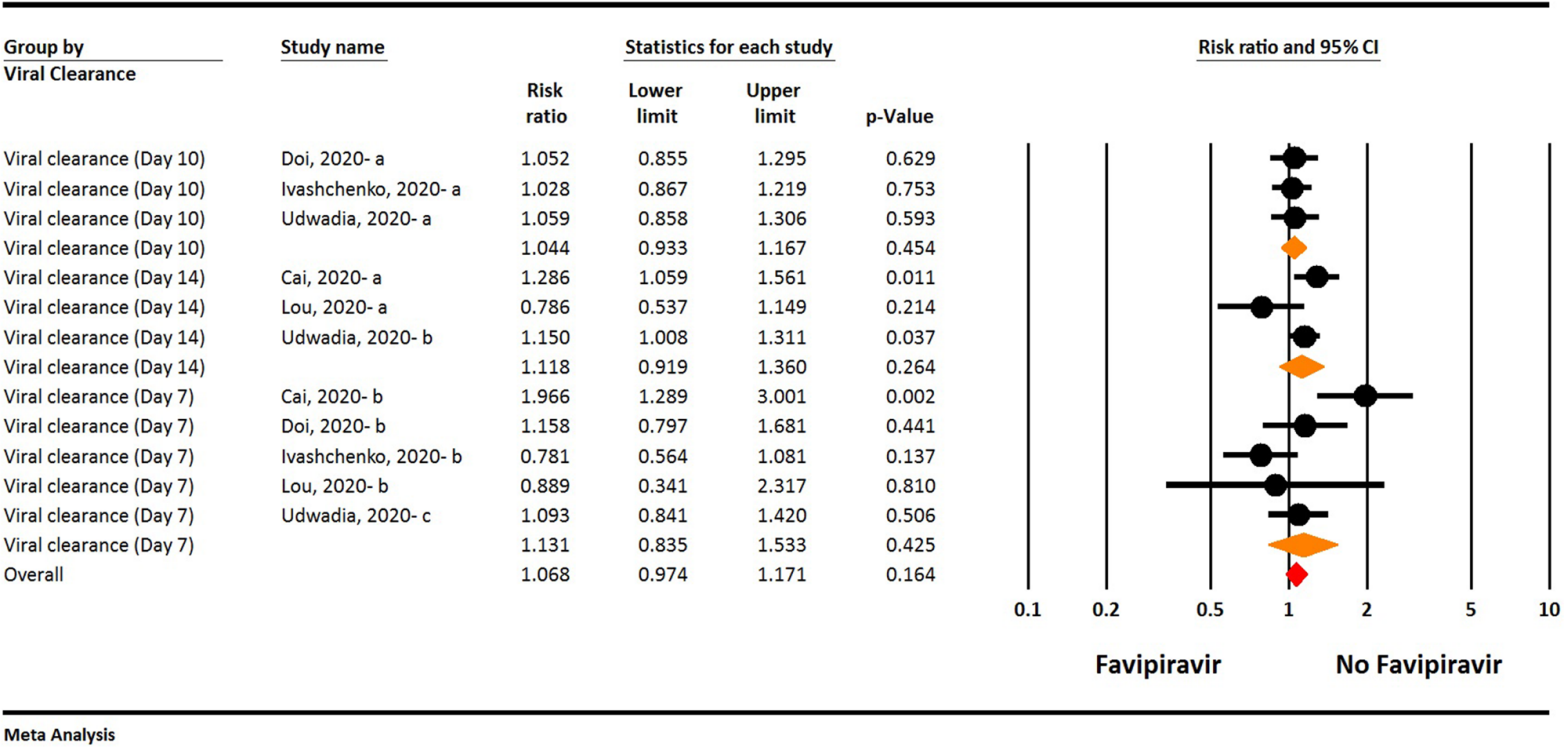
The updated meta-analysis of viral clearance of Favipiravir on COVID-19 patients after removal of Dabbous et al. data (Orange diamond: summary of sub groups; Red diamond: summary of total).

**Figure 2:**
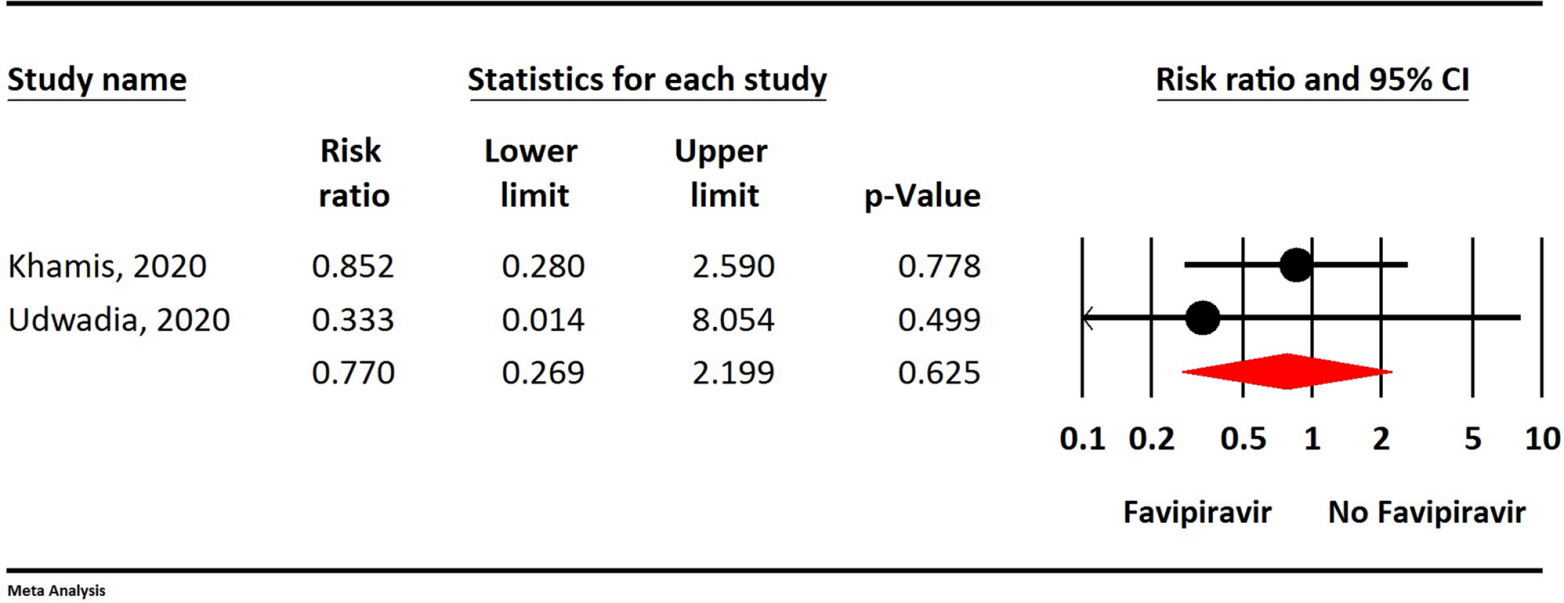
The updated meta-analysis of mortality of Favipiravir on COVID-19 patients after removal of Dabbous et al. data (Red diamond: summary of total).

**Viral clearance**

The result of the updated meta-analysis show that viral clearance 7, 10, and 14 days after hospitalization is not statistically different between the Favipiravir and control groups (RR = 1.13, 95% CI: 0.83-1.53; P = 0.425, I2 = 66.0%, P = 0.019 for 7 days; RR = 1.04, 95% CI: 0.93-1.16; P = 0.454, I2 = 0.0%, P = 0.973 for 10 days; RR = 1.11, 95% CI: 0.91-1.36; P = 0.264, I2 = 60.9%, P = 0.077 for 14 days) (Fig. 1).

**Mortality**

Based on the updated meta-analysis, the mortality rate in the Favipiravir group was approximately 23% lower than in the control group, but this finding is not statistically significant (RR = 0.77, 95% CI: 0.26-2.19; P = 0.625, I2 = 0.0%, P = 0.585) (Fig. 2).
