# Supplementary material for: The efficacy and safety of Favipiravir in treatment of COVID-19: a systematic review and meta-analysis of clinical trials
Source: Sci Rep. 2021 May 26;11:11022. doi: 10.1038/s41598-021-90551-6 (PMC8155021; doi:10.1038/s41598-021-90551-6)
Supplement: Supplementary file 1 — Supplementary Information. [file 41598_2021_90551_MOESM1_ESM.pdf]

Supplement 1: Proposed Search Strategy for PubMed

**Search**	**Search strategy**
**#1**	Search (("2019 nCoV" OR "2019 nCoV" OR "2019 novel coronavirus" OR "COVID-19" [Supplementary Concept] OR "severe acute respiratory syndrome coronavirus 2" [Supplementary Concept] OR "new coronavirus" OR "novel coronavirus" OR "SARS CoV-2" OR (Wuhan AND coronavirus) OR "SARS-CoV" OR "2019-nCoV" OR "SARS-CoV-2"))
**#2**	(favipiravir [Supplementary Concept] OR Avigan OR T-705 cpd)
**#3**	#1 AND #2
