# Supplementary material for: The efficacy and safety of Favipiravir in treatment of COVID-19: a systematic review and meta-analysis of clinical trials
Source: Sci Rep. 2021 May 26;11:11022. doi: 10.1038/s41598-021-90551-6 (PMC8155021; doi:10.1038/s41598-021-90551-6)
Supplement: Supplementary file 2 — Supplementary Figure. [file 41598_2021_90551_MOESM2_ESM.pdf]

	Random sequence generation (selection bias)	Allocation concealment (selection bias)	Blinding of participants and personnel (performance bias)	Blinding of outcome assessment (detection bias)	Incomplete outcome data (attrition bias)	Selective reporting (reporting bias)	Other bias
Cai et al/ 2020	−	−	−	+	+	+	+
Chen et al/ 2020	+	−	−	−	+	+	+
Dabbous et al/ 2020	+	+	−	−	+	+	+
Doi et al/ 2020	+	+	−	−	+	+	+
Ivashchenko et al/ 2020	+	+	−	−	+	?	+
Khamis et al/ 2021	+	?	−	−	+	+	+
Lou et al/ 2020	+	+	−	−	+	+	+
Udwadia et al/ 2020	+	+	−	−	+	+	+
Zhao et al/ 2021	+	+	−	−	+	+	+
