# Supplementary figures and images for: The efficacy and safety of Favipiravir in treatment of COVID-19: a systematic review and meta-analysis of clinical trials
Source: Sci Rep. 2021 May 26;11:11022. doi: 10.1038/s41598-021-90551-6 (PMC8155021; doi:10.1038/s41598-021-90551-6)

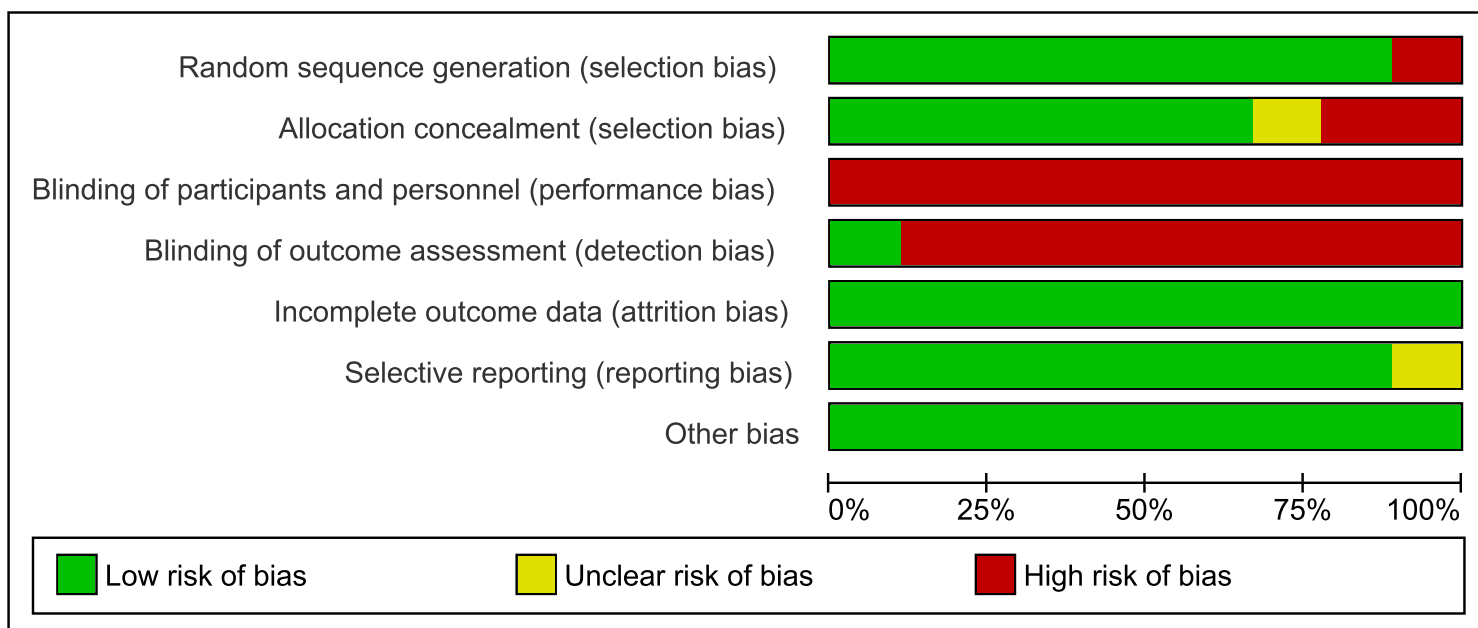

Supplement: Supplementary file 3 — Supplementary Figure. [file 41598_2021_90551_MOESM3_ESM.pdf]
